# Is the R&D Expenditure of Listed Companies Green? Evidence from China’s A-Share Market

**DOI:** 10.3390/ijerph191911969

**Published:** 2022-09-22

**Authors:** Boyu Li, Lishan Li, Tianlei Pi

**Affiliations:** School of Economics and Business Administration, Chongqing University, Chongqing 400030, China

**Keywords:** R&D, institutional investors, CO_2_ emission intensity, short-termism

## Abstract

Whether the company’s R&D expenditure has the green attribute is the focus of current environmental economics research. This paper empirically tests the relationship between R&D expenditure and CO_2_ emission intensity by taking Chinese A-share listed companies, from 2016 to 2020, as samples. The research found that the R&D expenditure of the company has a significant green effect of reducing its carbon dioxide emission intensity. Further research shows that the institutional investors play a mediating role in the relationship between R&D expenditure and CO_2_ emission intensity. And the “governance effect” of institutional investors is affected by “short-termism”, which leads to the “myopic” of enterprises’ management and urges them to invest in the short term, thus being detrimental to the company’s environmental performance. In addition, the green attribute of R&D expenditure only exists in the company which has a high concentration of institutional investors, indicating that the institutional investors possess the ability to identify the green value of R&D investment. Extended discussion shows that the investment of R&D personnel plays a moderating role in the first half path of the above mediating mechanism, which weakens the negative relationship between institutional investors and R&D investment. This paper provides empirical evidence for the government to improve environmental performance at the enterprise level. The results of this study show that, in order to reduce the CO_2_ emission intensity of enterprises, the government should improve incentives for enterprise R&D, make rational use of the information identification ability of institutional investors, advocate long-term investment philosophy, and strengthen the training of R&D team leaders and technicians.

## 1. Introduction

According to the Greenhouse Gas Bulletin released by the World Meteorological Organization (WMO) in 2019, carbon dioxide (CO_2_) in the atmosphere has reached a new level. The total warming effect of long-lived greenhouse gases increased by 43%, of which 82% was caused by CO_2_ [1]. Its long-term growth trend means that climate change will get worse in the future. The 2019 Emissions Gap Report, released by the United Nations Environment Programme (UNEP), points out that the current global emission reduction efforts are not enough, and the global CO_2_ emissions will not reach a peak by 2030 as is expected, which indicates the global warming trend will not be alleviated [2]. Reducing CO_2_ emissions and curbing global warming is the common responsibility of all mankind.

China, as the world’s largest carbon emitter, argues that it must shift the momentum of development. In 2015, The State Council issued a strategic document called Made in China 2025, which proposed to combine “innovation-driven” and “green development” to solve the contradiction between economic development and sustainable development [3]. Same as the developed countries, China is acknowledging the potential role of financial markets for environmental protection. In 2018, China established the ESG information disclosure framework for the first time and included it in the Governance Standards for Listed Companies. In 2020, after China proposed the dual carbon targets, the Guidelines on Promoting Investment and Financing in Response to Climate Change were issued. In the same year, public funds with the theme of “ESG investment” exceeded 100 billion yuan. In 2021, the Ministry of Ecology and Environment issued the Measures for the Management of Enterprise Environmental Information Disclosure in Accordance with Law and the Guidelines for the Format of Enterprise Environmental Information Disclosure in Accordance with Law. The pressure of policy and public opinion faced by listed companies are becoming more and more severe. However, based on the cost perspective, enterprises often lack the willingness to spend on environmental protection [4]. Neoclassical economic theory argues that corporate value is opposed to environmental protection because enterprise environmental protection expenditure will crowd out productive investment by increasing its cost, reducing profit [5].

The academic research on enterprises’ CO_2_ emission reduction mainly focuses on carbon trading and environmental regulation. In terms of carbon trading, the research results of different regional markets are inconsistent. Jong et al. (2014) believe that the EU carbon trading mechanism is conducive to enterprise emission reduction [6]. Nong et al. (2020) found, in their research on the Vietnamese market, that carbon trading can significantly curb carbon emissions [7]. Hu et al. (2020) demonstrated that carbon trading in China has a negative impact on the total factor productivity of enterprises, in which labor productivity is reduced and capital productivity is increased [8]. According to the research of Zhang et al. (2022), carbon trading in China only promotes emission reduction in high energy-consumption industry [9]. In terms of environmental regulation, it mainly starts from “Porter hypothesis”, which holds that environmental regulation has the innovation compensation effect, appropriate and reasonable environmental regulation can improve the efficiency of resource allocation, and is conducive to the improvement of technical level to achieve a win–win situation between environment and enterprises [10]. Timilsina et al. (2011) stated that carbon tax, if implemented simultaneously with subsidy policy, could alleviate the problem of reduced corporate income [11]. Song et al. (2018) concluded, through a large number of interviews, that Chinese enterprises’ carbon emission reduction is mainly affected by competitiveness and legitimacy [12]. Song et al. (2021) found that environmental regulation increases the preference for green investment of enterprises, thereby improving the carbon productivity [13]. You et al. (2022) demonstrated an “inverted U-shaped” relationship between environmental regulation intensity and enterprise green technology innovation [14].

Some scholars also explored the motivation of emission reduction in enterprises. Kent Walker et al. (2014) believe that enterprises can build positive reputation advantages through good environmental performance [15]. Cho et al. (2017) found that senior executives with academic experience pay more attention to fulfilling social responsibilities, based on their unique personal experiences, to form a self-restraint mechanism and promote enterprise green innovation [16]. Nie et al. (2022) and Zhao et al. (2019) explored the concern and influence of institutional investors on corporate environmental information disclosure [17,18]. Azar Jose et al. (2021) demonstrated that the world’s three largest asset management companies have a positive impact on the emission reduction in their portfolio companies [19].

It is generally believed that technological innovation is the key factor to deal with climate change and carbon emission reduction. Many scholars have discussed it from the macro level. Wang et al. (2017) took Chinese megacities as samples to study the impact of innovation in social and economic factors, urban form, and transportation network on carbon emissions, and they concluded that technological innovation can promote carbon emission reduction [20]. Töbelmann et al. (2020) proved that environmental innovation is conducive to reducing CO_2_ emissions in their analysis of the 27 EU countries [21]. Duan et al. (2022) pointed out that accelerating technological progress and industrial restructuring, as well as reducing energy consumption intensity of industrial sectors, could achieve CO_2_ emission peak faster [22]. 

Byrd et al. (2014) studied the determinants of corporate carbon emission reduction target setting and believed that the disclosure of absolute target was more compatible with the spirit of the Tokyo Protocol, but the use of intensity target was fairer to high-growth companies [23]. Zhou et al. (2022) expounded that carbon emission reduction is not achieved by reducing the absolute amount of carbon emissions but by reducing carbon intensity through measures such as economic restructuring and technological innovation [24]. Therefore, based on the perspective of economic benefits, this paper uses the enterprise CO_2_ emission intensity to measure the effect of R&D investment on carbon emission reduction.

The incremental contribution of this paper is as shown below. (1) At present, there are few studies about innovation investment and green development. This paper, as an exploration, empirically tests the relationship between R&D expenditure and CO_2_ emission intensity, providing a way of thinking for the research on enterprises’ response to climate change. (2) This paper further explores the role of institutional investors in corporate R&D activities on CO_2_ emission intensity, and it discovers the conduction mechanism of the impact of R&D on enterprises’ carbon emission reduction. It is helpful for enterprises and governments to grasp the specific path to improve the carbon emission reduction performance.

The following structure of this paper is as follows: The second part analyzes the theoretical mechanism of influence of R&D expenditure on CO_2_ emission intensity, the third part explains the variable selection, source, and model setting, the fourth part shows the empirical results and analyses, and the fifth part is the conclusion and policy enlightenment.

## 2. Theoretical Analysis and Research Hypothesis

### 2.1. CO_2_ Emission Intensity and R&D Expenditure

Knowledge is the key capital for modern enterprises to gain competitive advantages [25], innovation helps to improve the technological level and economic efficiency of companies, and enterprises aiming at profit maximization attach great importance to the positive impact of innovation input on corporate performance [26]. Tackling climate change, some scholars put forward the concept of “green innovation”, believing that green innovation can help reduce environmental pollution, emphasizing that enterprises can gain legitimacy through green innovation [27]. However, the process of green innovation is characterized by uncertainties, such as high investment and high risk, and the mismatch of environmental regulation and government subsidy measures will reduce the enthusiasm of enterprises for green innovation [28].

There is no denying that, for enterprises, the green innovation helps to reduce the negative externality effect in future production activities as well as to achieve sustainable development. However, the academic community has not reached a unified standard on the definition of green innovation. The purpose of enterprise innovation activities is to improve financial performance and achieve a competitive advantage. The research shows that technological innovation can minimize the environmental burden of industrial ecological boundary [29], improve the efficiency and sustainability of resource utilization [30], and reduce energy consumption intensity [31], which is helpful for controlling CO_2_ emissions.

Nowadays, environmental issues are receiving widespread attention, and enterprises are motivated to break the boundary of traditional advantages and achieve green development. It would not only enhance the reputation value of enterprises but also bring significant competitive advantages to enterprises, as investors and consumers pursue green enterprises [32]. Therefore, it can be considered that the innovation investment of enterprises is green. Due to the current A-share market, many listed companies do not disclose their R&D personnel; therefore, this paper puts forward the following hypotheses:

**Hypothesis** **1.** *R&D investment contributes to the reduction in CO_2_ emission intensity.*

### 2.2. The Mediating Role of Institutional Investors

Financial markets have a direct impact on the function and value of businesses, and they play an important role in promoting emission reduction and sustainable development [33,34]. Corporate environmental behavior can bridge the interest differences between corporate shareholders and stakeholders, and align the long-term interests [35]. If an enterprise has environmental problems, not only does the enterprise itself have environmental liability risks but also the reputation of its stakeholders will also be affected [36].

As professional investors with abundant funds, institutional investors can not only identify the real information and risk factors from the public information of enterprises [37,38] but also obtain the real information of enterprises through research and other ways [39]. Institutional investors are market participants with information advantages and could quickly find valuable investment targets. In particular, institutional investors can exert a “governance effect” through the “exit threat” [40]. This means that institutional investors can influence the governance of their portfolio companies in order to achieve environmental objectives and gain investment benefits.

However, some scholars pointed out the ideas of individualism, profit maximization, and economic rationality existed in the financial sector [41,42]. This would lead investors to pay more attention to the realization of self-interest and choose rational—rather than reasonable—behavior in the financial market [43], which suggests that the “governance effect” brought by institutional investors on their portfolio companies may generally be negative. Laverty (1996) proposed “short-termism” and believed that focusing on short-term returns was the dominant logic in the financial market [44]. Studies have shown that, in the context of the prevalence of short-termism, the complete change cycle of a portfolio is now significantly lower than 50 years ago; more extreme, some institutional investors emphasize the use of quantitative high-frequency trading strategy arbitrage [45], and such “short-termism” puts enormous pressure on corporate behavior. On the one hand, investors (stakeholders) require enterprises to achieve short-term benefits [46], while on the other hand, the high liquidity in the stock market increases the risk of being acquired [47]. Management has been forced into “myopic” behavior, leading to the abandonment of long-term investment projects in favor of more traditional and moderate ones [48]. Thus, it has a negative impact on corporate governance and hinders the CO_2_ emission reduction process of enterprises.

In short, institutional investors will be attracted by the innovation value of enterprises and have an impact on the emission reduction in enterprises through the “governance effect”. Therefore, we propose the following hypothesis:

**Hypothesis** **2.** *R&Dexpenditure influences CO_2_ emission intensity through the mediating effect of institutional investor*.

## 3. Research Design

### 3.1. Sample Selection and Data Sources

This paper selects A-share listed companies covered by Trucost (a commercial provider of corporate carbon emission data), in the period between 2016 and 2020, as research samples, excluding the companies which have missing main data and are marked with ST or ST*. The CO_2_ emission data are from the Trucost database, the annual turnover rate is from CSMAR database, the shareholding data of institutional investors are from Eastern Wealth Choice database, and other data are from WIND database. Excel 2019 was used for data collation, and STATA 16 was used for data analysis.

### 3.2. Variable Definitions

#### 3.2.1. Explained Variable

CO_2_ Emission Intensity (CEI). CO_2_ emissions from resources owned or controlled by the company (classified under the GHG Agreement) are calculated by dividing the company’s revenue.

#### 3.2.2. Explanatory Variables

R&D expenditure (RD). It is calculated by dividing the amount of R&D expenditure by the company’s revenue.

#### 3.2.3. Mediating Variable

Institutional Investors (II). Shareholding ratio of institutional investors, which included public fund, private placement fund, social security fund, Qualified Foreign Institutional Investor (QFII), insurance, securities trader, trust institution, and other investment companies. It represents the extent to which institutional investors can exert influence on corporate management.

#### 3.2.4. Moderator

R&D Personnel (RP). It is calculated by dividing the technical staff by the total employees.

#### 3.2.5. Control Variables

Based on theoretical analysis, this paper takes Turnover Rate (TR), used as a measure of liquidity, it is the sum of the turnover rates for each trading day of the year. Refer to the study by Azar José et al. (2021), this paper also takes Debt-to-assets ratio (DTA), non-current assets to total assets ratio (NTA), total assets (asset), and return on assets (ROA) as control variables [19]. The details are shown in Table 1. In addition, this paper also added the region (province) and industry (industry) dummy variable. For province, if the company is located in the region where the carbon emission trading pilot is carried out, the value is set to 1; otherwise, the value is 0. For  industry, the value for manufacturing industry is 1; otherwise, the value is 0.

### 3.3. Model

#### 3.3.1. R&D Expenditure and CO_2_ Emission Intensity

Generally, this study should consider the cross-section fixed effect. However, due to the limitations of the sample, where the number of objectives is much larger than the number of years, if we controlled the cross-section fixed effect, the loss of degree of freedom would be large. In that case, we control the time, province, and industry fixed effects, minimizing the bias caused by omitted variables as much as possible. Thus, we use the Least Squares Dummy Variable (LSDV) to study the relationship between R&D expenditure and CO_2_ emission intensity, estimating the following model:

(1) $$CEI_{i,t}=\beta_{0}+\beta_{1}RD_{i,t-1}+\beta_{2}TR_{i,t}+\beta_{3}DTA_{i,t-1}+\beta_{4}DTA_{i,t-1}+\beta_{5}NTA_{i,t-1}\;\;\;\;\;\;\;\;\;\;\;\;\;\;\;\;\;+\beta_{6}asset_{i,t-1}+\beta_{7}ROA_{i,t-1}+\gamma_{t}+\delta province_{i}+\theta industry_{i}+\varepsilon_{i,t}$$

where γ denotes year fixed effects, province denotes province fixed effects, and industry denotes industry fixed effects. Subindexes i and t refer to firm i and year t.

#### 3.3.2. The Mediating Effect of Institutional Investors

**Figure 1:**
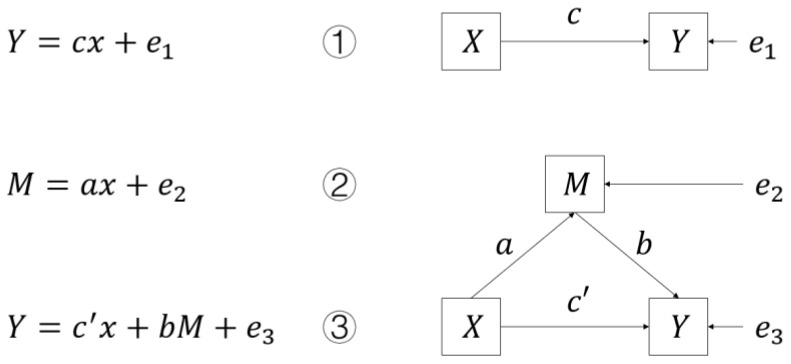
Illustration of the mediating effect model.

To test whether R&D expenditure influences CO_2_ emission intensity through the mediating effect of institutional investors, on the basis of the benchmark model, we further establish the following recursive model referring to the analysis method of Wen et al. (2004) [49]. The method is illustrated in Figure 1:

First of all, we are interested in the effect of X on Y. As shown in Equation (1), if the regression coefficient c is significant, we would like to consider the mediating variable M or, otherwise, stop the analysis. In the Equations (2) and (3), if regression coefficient a and regression coefficient b are significant, it means that at least part of the influence of X on Y is realized through the mediating variable M. If the regression coefficient c’ is significant, it indicates that there is a partial mediating effect; If the regression coefficient c’ is not significant, it indicates that there is a full mediating effect, that is, X has the influence on Y through M.

Therefore, the following recursive model is established:

(2) $$II_{i,t}=\beta_{0}+\beta_{1}RD_{i,t-1}+\beta_{2}TR_{i,t}+\beta_{3}DTA_{i,t-1}+\beta_{4}DTA_{i,t-1}+\beta_{5}NTA_{i,t-1}+\beta_{6}asset_{i,t-1}\;+\beta_{7}ROA_{i,t-1}+\gamma_{t}+\delta province_{i}+\theta industry_{i}+\varepsilon_{i,t}\;\;$$

(3) $$CEI_{i,t}=\beta_{0}+\beta_{1}II_{i,t}+\beta_{2}RD_{i,t-1}+\beta_{3}TR_{i,t}+\beta_{4}DTA_{i,t-1}+\beta_{5}DTA_{i,t-1}+\beta_{6}NTA_{i,t-1}\;\;\;\;\;\;\;\;\;\;\;+\beta_{7}asset_{i,t-1}+\beta_{8}ROA_{i,t-1}+\gamma_{t}+\delta province_{i}+\theta industry_{i}+\varepsilon_{i,t}\;$$

## 4. Empirical Results and Analysis

### 4.1. Descriptive Statistics and Correlation Analysis

#### 4.1.1. Descriptive Statistics and Analysis of Variables

There are 153 companies in the sample, and the number and industry distributions are shown in the Table 2. We can learn from the Table 2 that most companies are distributed in the manufacturing industry.

We can learn from Table 3 that the coefficient of variation of CO_2_ Emission Intensity (CEI), R&D expenditure (RD), and institutional investors (II) are relatively large, which means the main variables are quite different. In terms of control variables, there are also obvious differences in other variables except total assets (asset). The mean value of province is 0.392, indicating that 39.2% of the listed companies in the sample are located in pilot areas of carbon emission trading.

#### 4.1.2. Correlation Analysis

As shown in Table 4, the upper triangle is the Spearman’s correlation coefficient and significance level, while the lower triangle is the Pearson’s coefficient and significance level. The Spearman correlation coefficient and the Pearson correlation coefficient of CO_2_ emission intensity (CEI) and R&D expenditure (RD) are negative, and they are significant at 1% level, which indicates that the higher the R&D expenditure, the lower the CO_2_ emission intensity. The Spearman correlation coefficient and the Pearson correlation coefficient of institutional investors (II) and R&D expenditure (RD) are negative, and they are significant at 1% level, which indicates that the larger the R&D expenditure, the lower the degree of institutional investors’ participation in corporate management. The Spearman correlation coefficients of CO_2_ emission intensity (CEI) and institutional investors (II) are positive but not significant; however, the Pearson correlation coefficient of them is positive and significant at 1% level, which indicates that the participation of institutional investors in corporate management is positively related to the level of CO_2_ emissions intensity.

Table 5 shows the variance inflation factor (VIF) of the variables. Among them, the maximum VIF value was 2.13, and the VIF of all variables was far less than 10, indicating that there was no serious multicollinearity problem among the table variables.

### 4.2. Analysis of Regression Results

Table 6 shows the regression results of the main effect and mediating effect. We used the three-step test method of the mediating effect of Wen et al. (2004), for reference, to analyze the regression results [49].

In column (1) of Table 6, model (1) is used to test the impact of R&D expenditure on CO_2_ emission intensity of listed companies. The regression results show that R&D expenditure is negatively correlated with CO_2_ emission intensity at the 1% significance level. The regression coefficient is −2.061, indicating that R&D expenditure is green, and each 1% increase in R&D expenditure reduces CO_2_ emission intensity by 2.061%, which is in line with Hypothesis 1.

Columns (2) and (3) of Table 6 show the subsequent mediating effect test, and model (2) studies how R&D expenditure affects the institutional investors. The regression results show that R&D expenditure is negatively correlated with institutional investors at the 1% significance level, and the regression coefficient is −0.507, indicating that the shareholding ratio of institutional investors decreases by 0.507%, with each having a 1% increase in R&D expenditure. In the next step, model (3) tests whether the institutional investors play a mediating role between CO_2_ emission intensity and R&D expenditure. In the regression results, the institutional investors are positively correlated with CO_2_ emission intensity at the significance level of 1%, indicating the existence of a mediating effect. At 1% significance level, R&D expenditure is negatively correlated with CO_2_ emission intensity, and the regression coefficient is −1.741, which is greater than that in model (1), indicating that the institutional investors play a part of the mediating role. As analyzed in Hypothesis 2 above, the “short-termism” of institutional investors weakens the ability of R&D expenditure to reduce CO_2_ emission intensity.

### 4.3. Robustness

#### 4.3.1. Change the Core Variable

The explained variable CO_2_ emission intensity is replaced by the absolute value of CO_2_ emission ($CO_{2}$), and the unit is equivalent to 10 k tons of CO_2_. The results are shown in Table 7, and column (1) is listed as the benchmark regression. The results show that R&D expenditure is negatively correlated with CO_2_ emission intensity at the significance level of 1%, and the regression coefficient is −3.004, which is consistent with the above conclusion. Columns (2) and (3) are the subsequent mediating effect test, column (3) shows that institutional investors is positively correlated with carbon dioxide emissions at the significance level of 1%, and the R&D expenditure is negatively correlated with CO_2_ emission intensity at the significance level of 5%, with a regression coefficient of −2.271. The conclusion is consistent with the above, indicating the robustness of the benchmark regression in this paper.

#### 4.3.2. Regression Based on Group

This paper performs grouped regression on the samples, and the results are shown in Table 8. Most of the listed companies in the sample belong to the manufacturing industry, so the sample is classified by industry: column (1) represents the manufacturing industry group and column (2) represents other industries group. Then, taking 50% of the shareholding ratio of institutional investors as the critical value, the sample is divided into two groups (high concentration of institutional investment group and low concentration of institutional investment group), and the results are shown in columns (3) and (4), respectively. The results all show that R&D expenditure is green and can promote the reduction in CO_2_ emission intensity, which is consistent with the above conclusion.

Particularly, the regression coefficient of RD in column (1) is −4.998, which is smaller than that in column (2) −2.077, indicating that the effect of R&D expenditure on reducing CO_2_ emission intensity is more obvious in manufacturing industry. Moreover, the regression coefficient of RD in column (3) is −3.617, which is consistent with the above conclusion. What is noteworthy is that, in column (4), we surprising found that, although the regression coefficient of RD is −0.226, the result was not significant, which may suggest there is heterogeneity, as well as indicate that the institutional investors have advantages identifying the green value of R&D expenditure, which is why they held more stocks of these companies with environmental friendly R&D investment.

#### 4.3.3. Endogeneity

Considering the possible endogeneity problems, such as reverse causality. In the competitive market, enterprises have to carefully examine the risk of R&D investment due to the continuous dynamic game, and enterprises will adopt various technological development strategies according to their different positions in the competition [50]. Therefore, in the context of emphasis on emission reduction, the different CO_2_ emission intensities of enterprises may affect their relevant R&D investments and directions.

In this paper, the Two Stage Least Square (TSLS) is used to test the benchmark regression. This paper selects two instrumental variables: ① operating income (income), which represents an enterprise’s scale and market conditions. Only with better financial performance can it carry out R&D activities better. Therefore, an enterprise’s income can effectively affect its R&D investment. ② Average institutional investors’ shareholding ratio of the industry (avg_ratio). Speculative institutional investors may affect the shareholding ratio of institutional investors in a single company due to short-term news, but it is difficult to affect the average institutional investor’s shareholding ratio of the industry. Many studies have proved that institutional investors with long-term investment philosophy tend to invest in companies with good innovation performance, so the average institutional investor’s shareholding ratio of the industry reflects the R&D investment level that enterprises should have reached to a certain extent. To sum up, enterprise operating revenue and average institutional shareholding ratio of the industry meet the requirements of correlation and endogeneity of instrumental variables.

Table 9 shows the results of the regression. Column (1) is the above LSDV regression results. Column (2) is the first stage estimation, in which the regression coefficient of the instrumental variable  income is −1.629 and significant at the 1% level, that is, the higher the operating income, the lower the CO_2_ emission intensity of the enterprise, which is consistent with the expectation. The regression coefficient of the instrumental variable avg_ratio is −0.244 and significant at 1% level, that is, the higher the average institutional investor’s shareholding ratio of the industry, the lower the CO_2_ emission intensity of enterprises, which is also consistent with expectations. In addition, the Kleibergen–Paap rk LM test significantly rejects the null hypothesis, indicating that there is no underidentification problem, that is, the selected instrumental variables are correlated with the endogenous explanatory variables. The Cragg–Donald Wald F statistic and Kleibergen–Paap rk Wald F statistic are both larger than the critical value of Stock–Yogo weak instrumental variable test, which significantly rejects the null hypothesis of the weak instrumental variable, indicating that there is no weak instrumental variable problem. Hansen’s J test also shows that the two instrumental variables selected in this paper are appropriate. Column (3) is the second stage estimation, and the results show that the regression coefficient of RD is −11.188 at 1% significant level, indicating that R&D investment could reduce the CO_2_ emission intensity, which is consistent with the above conclusion.

### 4.4. Extended Discussion

As a part of R&D input, R&D personnel should be as meaningful as R&D expenditure is. The productivity of different R&D teams varies due to factors such as structure, size, and culture [51,52,53], which will inevitably affect the utilization efficiency of R&D expenditure and further affect the CO_2_ emission of enterprises. Since China’s A-share listed companies rarely publish the number of R&D personnel, most of them only publish the number of technicians, so we consider whether it has a biased effect on CO_2_ emission intensity. The amount of technicians is used to represent the R&D personnel to discuss this idea.

Referring to the research method of Li et al. (2021) [54], the number of R&D personnel is taken as the moderator or “mediated moderator“. This concept was mentioned by Baron et al. (1986) [55], and Wen et al. (2006) made an analysis on it in detail [56]. The specific model is as follows:

(4) $$CEI_{i,t}=\beta_{0}+\beta_{1}RD_{i,t-1}+\beta_{2}RP_{i,t-1}+\beta_{3}RD_{i,t-1}*RP_{i,t-1}+\beta_{4}TR_{i,t}+\beta_{5}DTA_{i,t-1}\;+\beta_{6}DTA_{i,t-1}+\beta_{7}NTA_{i,t-1}+\beta_{8}asset_{i,t-1}+\beta_{9}ROA_{i,t-1}+\gamma_{t}\;+\delta pprovince_{i}+\theta industry_{i}+\varepsilon_{i,t}$$

(5) $$II_{i,t}=\beta_{0}+\beta_{1}RD_{i,t-1}+\beta_{2}RP_{i,t-1}+\beta_{3}RD_{i,t-1}*RP_{i,t-1}+\beta_{4}TR_{i,t}+\beta_{5}DTA_{i,t-1}\;+\beta_{6}DTA_{i,t-1}+\beta_{7}NTA_{i,t-1}+\beta_{8}asset_{i,t-1}+\beta_{9}ROA_{i,t-1}+\gamma_{t}\;+\delta province_{i}+\theta industry_{i}+\varepsilon_{i,t}\;$$

(6) $$CEI_{i,t}=\beta_{0}+\beta_{1}RD_{i,t-1}+\beta_{2}II_{i,t}+\beta_{3}RP_{i,t-1}+\beta_{4}RD_{i,t-1}*RP_{i,t-1}+\beta_{5}TR_{i,t}\;+\beta_{6}DTA_{i,t-1}+\beta_{7}DTA_{i,t-1}+\beta_{8}NTA_{i,t-1}+\beta_{9}asset_{i,t-1}+\beta_{10}ROA_{i,t-1}\;+\gamma_{t}+\delta province_{i}+\theta industry_{i}+\varepsilon_{i,t}\;\;$$

**Figure 2:**
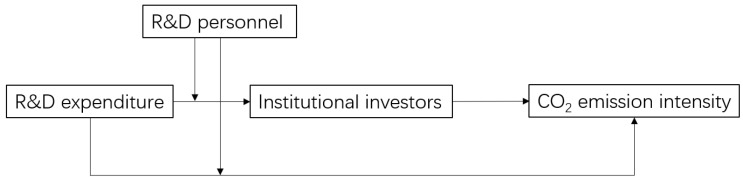
Logic diagram about the first half path of mediated moderating effect.

Model (4) tests the moderating effect of R&D personnel on R&D expenditure and CO_2_ emission intensity. When the regression coefficient of the intersection term RD∗RP is significant, it indicates that R&D personnel has an obvious moderating effect on the relationship between R&D expenditure and CO_2_ emission intensity. Models (5) and (6) test the moderating effect of R&D personnel on the first half path of the relationship between R&D expenditure and CO_2_ emission intensity. When the regression coefficient of the interaction term RD∗RP, in model (5), is significant and the regression coefficient of the institutional investors in model (6) is significant, indicating that the moderating effect exists in the first half path of the mediation model. The logic diagram is shown in Figure 2, and the results are shown in Table 10.

As shown in column (1) of Table 10, which shows the results of model (4), the regression coefficient of the intersection term, RD∗RP, is significantly positive at the 1% level, indicating that R&D personnel plays a moderating role on R&D expenditure and CO_2_ emission intensity, and the input of R&D personnel weakens the negative relationship between R&D expenditure and CO_2_ emission intensity. The possible explanation is that the raising number of R&D personnel or the expansion of R&D team size would reduce the R&D efficiency to some extent.

As shown in column (2), which shows the results of model (5), the regression coefficient of the interaction term, RD∗RP, is significantly positive at the 5% level, indicating the size of R&D team is preferred by institutional investors, and R&D personnel weakens the negative correlation between the R&D expenditure and the institutional investors. The results of model (6) are shown in column (3), and the regression coefficient of ratio is significantly positive at 1% level, indicating that the moderating effect exists in the first half path of the moderated mediating effect model, which means that part of the mediating effect of institutional investors on the relationship between R&D expenditure and CO_2_ emission intensity is moderated by R&D personnel. In other words, the R&D personnel can not only weaken the negative relationship between the R&D expenditure and the institutional investors but also weaken the negative relationship between the investment of R&D expenditure and CO_2_ emission intensity.

On the one hand, with the continuous growing number of R&D personnel, institutional investors, who tend to take short-term profits from news, may increase their holdings, promoting the “myopic” behavior of corporate executives. In turn, it leads to poor emission reduction performance of enterprises. On the other hand, the size of the R&D team may increase, which reduces the working efficiency of the team, leads to the decline of innovation efficiency, and finally makes the emission reduction performance of the enterprise poor.

## 5. Conclusions

Based on the data of A-share listed companies in China from 2016 to 2020, this paper empirically analyzes the relationship between R&D expenditure and CO_2_ emission intensity. The results show that: (1) R&D expenditure has the green attribute, and its increase will significantly reduce the CO_2_ emission intensity of listed companies; (2) the institutional investors play a partial mediating role between R&D expenditure and CO_2_ emission intensity; (3) the evidence that R&D expenditure is green is stronger in manufacturing than other industries; (4) the green attribute of R&D expenditure only exists in the listed companies with a high concentration of institutional investors, indicating that institutional investors have the ability to identify the potential environmental benefits of the R&D project, but they are reluctant to take the risks associated with the new and long-term R&D investments; (5) R&D personnel play a moderating role in the mechanism by which R&D expenditure reduces CO_2_ emission intensity.

Based on the research conclusions, this paper puts forward the following suggestions: Firstly, the government should establish an innovation support mechanism and increase subsidies for the R&D expenditure of enterprises. As the regulator in economic activities, the government has the responsibility and ability to properly intervene in enterprise activities, encourage innovative activities, and pay attention to the possibility of green benefits brought by innovative activities. Secondly, the government, financial intermediaries, and the media should strengthen the publicity of enterprises with good performance in environmental governance, as well as encourage listed companies to develop their innovation and R&D activities to green development. Thirdly, the whole society needs a long-term investment education campaign, urging the financial sector to abandon “short-termism”, emphasizing the benefits that the environmental and social responsibility of corporate can bring, and focusing on long-term investment returns. Fourthly, enterprises should strengthen the training of senior executives and focus on preventing the occurrence of the “myopic” behavior of senior executives. Fifth, enterprises should properly control the size of R&D teams, strengthen the training of R&D personnel, and improve the professional quality of them.

This paper is a preliminary exploration of CO_2_ emissions reduction at the enterprise level. As China’s environmental governance started relatively late, this paper can provide reference for countries that are lagging behind in environmental governance. Since there are few studies about such topic, this study also enriches present researches. However, this paper has the following limitations. First of all, at present, China hasn’t built up the carbon emission accounting system, and the environmental governance data disclosure system of listed companies has just begun to be implemented. The majority of listed companies did not disclose their CO_2_ emission data, so the data used in this article is from the Trucost database and can be obtained from Wharton Research Data Services (WRDS). According to the user manual, all data are estimated through enterprise activities rather than officially released. Besides, this study contains a few of the listed companies (only about 3% of China’s A-share listed companies), and the time span is only five years, which limits the depth of research. When the environmental information disclosure system of China’s listed companies is built up, this study can be further improved by using other analytical methods, such as Time Series, Cluster Analysis, and so on.

## Figures and Tables

**Table 1:** Variable definition and source.

Variable Types	Variable Name	Symbol	Definition	Sources
Explained variable	CO_2_ Emission Intensity	CEI	Logarithm of CO_2_ Emission Intensity	Trucost
Explanatory variables	R&D expenditure	RD	R&D expenditure as a percentage of the company’s total revenue	Wind
Mediating variable	Institutional investors	II	Shareholding ratio of institutional investors	Eastern Wealth Choice
Moderator	R&D Personnel	RP	The number of technicians divided by the total number of employees in the company	Wind
Control variables	Turnover Rate	TR	Turnover Rate	CSMAR
	Debt-to-assets ratio	DTA	Debt-to-assets ratio	Wind
	Non-current assets to total assets ratio	NTA	Non-current assets to total assets ratio	Wind
	Total assets	asset	Total assets	Wind
	Return on assets	ROA	Return on assets	Wind

**Table 2:** The number of companies and their industry distribution.

Variable	Number	Proportion
Mining industry	6	3.92%
Electricity, heat, gas and water production and supply	2	1.31%
Real estate	1	0.65%
Construction industry	4	2.61%
Financial industry	2	1.31%
Scientific research and technology services	2	1.31%
Agriculture, forestry, animal husbandry and fishery	1	0.65%
Wholesales and retail trade	1	0.65%
Water conservancy, environment and public utilities management	1	0.65%
Culture, sports and entertainment	1	0.65%
Information transmission, software and information technology services	12	7.84%
Manufacturing industry	119	77.8%
Comprehensive industry	1	0.65%

**Table 3:** Descriptive statistics of variables.

Symbol	Obs.	Mean	Std.	CV	Min	Max
CEI	765	30.391	84.365	2.776	0.102	493.635
RD	765	4.530	5.149	1.137	0.006	48.476
II	765	48.396	21.534	0.445	0.012	92.731
RP	765	15.425	14.854	0.963	0.366	79.858
TR	765	21.250	494.462	23.269	0.147	13679.33
DTA	765	45.807	18.384	0.401	4.530	111.779
NTA	765	43.752	18.877	0.431	1.368	92.545
asset	765	23.437	1.144	0.049	21.223	28.341
ROA	765	5.614	7.497	1.335	−77.472	37.252
province	765	0.392	0.489	1.246	0	1

**Table 4:** Spearman and Pearson correlation coefficient test.

	CEI	RD	RP	II	TR	DTA	NTA	Asset	ROA
CEI	1.000	−0.398 ***	−0.464 ***	0.029	−0.030	0.007	0.454	0.119	0.068
RD	−0.175 ***	1.000	0.641 ***	−0.183 ***	0.148 ***	−0.138 ***	−0.219 ***	−0.251 ***	0.006
RP	−0.225 ***	0.684 ***	1.000	−0.216 ***	0.211 ***	−0.093 ***	−0.293 ***	−0.290 ***	−0.014
II	0.145 ***	−0.170 ***	−0.217 ***	1.000	−0.480 ***	0.003	−0.027	0.238 ***	0.127 ***
TR	−0.010	−0.027	−0.027	0.024	1.000	0.038	0.023	−0.252 ***	−0.159 ***
DTA	0.107 ***	−0.134 ***	−0.134 ***	0.014	−0.041	1.000	0.075 **	0.552 ***	−0.478 ***
NTA	0.327 ***	−0.149 ***	−0.149 ***	−0.015	−0.033	0.061 *	1.000	0.181 ***	−0.148 ***
asset	0.123 ***	−0.245 ***	−0.245 ***	0.268 ***	−0.030	0.515 ***	0.144 ***	1.000	−0.191 ***
ROA	−0.025	0.018	−0.039	0.141 ***	0.035	−0.394 ***	−0.146 ***	−0.092 **	1.000

Note: ***, ** and * indicate that the coefficient significance test has passed at the significance level of 1%, 5% and 10%, respectively.

**Table 5:** VIF of variables.

Variable	VIF	1/VIF
RD	1.91	0.524
RP	2.25	0.445
II	1.18	0.846
TR	1.01	0.989
DTA	1.70	0.590
NTA	1.13	0.883
asset	1.78	0.563
ROA	1.29	0.778
province	1.13	0.884
industry	1.19	0.839

**Table 6:** The benchmark regression and mediating effect results.

Variable	(1) CEI	(2) II	(3) CEI
RD	−2.061 *** (−4.61)	−0.507 *** (−3.30)	−1.741 *** (−4.43)
II			0.632 *** (3.84)
TR	−0.001 ** (−2.25)	0.001 *** (5.83)	−0.002 *** (−3.18)
DTA	0.341 * (1.90)	−0.109 ** (−2.22)	0.409 ** (2.21)
NTA	1.237 *** (8.55)	−0.043 (−1.15)	1.264 *** (8.54)
asset	2.683 (1.19)	5.296 *** (6.94)	−0.666 (−0.26)
ROA	0.604 * (1.70)	0.357 *** (3.29)	0.379 (1.08)
Constant	−67.106 (−1.27)	−79.917 *** (−4.39)	−19.105 (−0.33)
Time fixed effect	Controlled	Controlled	Controlled
Province fixed effects	Controlled	Controlled	Controlled
Industry fixed effects	Controlled	Controlled	Controlled
Obs.	765	765	765
$R^{2}$	0.1742	0.1428	0.1965

Note: “ robust T-statistic” is enclosed in parentheses; *, **, and *** denote the levels of significance by 10%, 5%, and 1%, respectively.

**Table 7:** Regression results after changing the core explanatory variables.

Variable	(1) $CO_{2}$	(2) II	(3) $CO_{2}$
RD	−3.004 *** (−2.83)	−0.507 *** (−3.30)	−2.271 ** (−2.32)
II			1.447 *** (2.77)
TR	−0.001 (−0.41)	0.001 *** (5.83)	−0.002 (−1.26)
DTA	1.744 ** (2.49)	−0.109 ** (−2.22)	1.902 *** (2.62)
NTA	2.729 *** (5.12)	−0.043 (−1.15)	2.791 *** (5.19)
asset	50.450 *** (4.66)	5.296 *** (6.94)	42.787 *** (3.77)
ROA	1.690 (1.08)	0.357 *** (3.29)	1.174 (0.77)
Constant	−1264.27 *** (−5.19)	−79.917 *** (−4.39)	−1174.436 *** (−4.66)
Time fixed effect	Controlled	Controlled	Controlled
Province fixed effects	Controlled	Controlled	Controlled
Industry fixed effects	Controlled	Controlled	Controlled
Obs.	765	765	765
$R^{2}$	0.1341	0.1428	0.1430

Note:“ robust T-statistic” is enclosed in parentheses; **, and *** denote the levels of significance by 5%, and 1%, respectively.

**Table 8:** Subsample regression results.

Variable	(1) CEI	(2) CEI	(3) CEI	(4) CEI
RD	−4.998 *** (−5.08)	−2.077 *** (−3.26)	−3.617 *** (−4.05)	−0.226 (−1.40)
TR	−0.001 ** (−2.33)	−2.987 (−1.22)	−0.002 ** (−2.05)	−1.331 ** (−2.54)
DTA	0.173 (0.88)	0.368 (0.72)	1.034 ** (5.57)	−0.184 (−1.55)
NTA	1.271 *** (7.96)	0.839 *** (2.93)	1.826 *** (6.46)	0.761 *** (5.33)
asset	8.160 *** (3.10)	−9.724 * (−1.89)	0.898(0.23)	2.795(1.32)
ROA	0.135 (0.28)	0.802 (1.43)	1.140 (1.00)	0.514 ** (2.44)
Constant	−199.348 *** (−3.58)	274.464 ** (2.28)	−20.443 (−0.23)	−78.458 (−1.66)
Time fixed effect	Controlled	Controlled	Controlled	Controlled
Province fixed effects	Controlled	Controlled	Controlled	Controlled
Industry fixed effects			Controlled	Controlled
Obs.	595	170	373	392
$R^{2}$	0.2075	0.1999	0.2938	0.2122

Note:“ robust T-statistic” is enclosed in parentheses; *, **, and *** denote the levels of significance by 10%, 5%, and 1%, respectively.

**Table 9:** Regression results of Two Stage Least Square (TSLS).

Variable	(1) CEI	(2) CEI	(3) CEI
Estimation method	LSDV	First stage of TSLS	Second stage of TSLS
RD	−2.061 *** (−4.61)		−11.188 *** (−3.15)
income		−1.629 *** (−5.77)	
avg_ratio		−0.244 *** (−2.85)	
Control variable	Controlled	Controlled	Controlled
Time fixed effect	Controlled	Controlled	Controlled
Province fixed effects	Controlled	Controlled	Controlled
Industry fixed effects	Controlled	Controlled	Controlled
Obs.	765	765	765
F test of included instruments (*p* value)		54.29 (0.000)	
Kleibergen–Paap rk LM statistic (*p* value)			38.667 (0.000)
Cragg–Donald Wald F statistic			25.757
Kleibergen–Paap rk Wald F statistic			21.708
Stock-Yogo weak ID test critical values: 10% maximal IV			19.93
Hansen’s J statistic (*p* value)			0.212 (0.645)

Note:“ robust T-statistic” is enclosed in parentheses of Column (1) and (2);” z-statistic” is enclosed in parentheses of Column (3); *** denote the levels of significance by 1%.

**Table 10:** Regression results of the mediated moderating effect.

Variable	(1) CEI	(2) II	(3) CEI
RD	−5.659 *** (−4.23)	−0.834 ** (−2.23)	−5.185 *** (−4.06)
II			0.568 *** (3.62)
RP	−1.121 *** (−4.55)	−0.307 *** (−3.97)	−0.946 *** (−4.21)
RD∗RP	0.099 *** (3.86)	0.015 ** (2.28)	0.090 *** (3.64)
TR	−0.002 *** (−3.72)	0.001 *** (4.02)	−0.003 *** (−4.14)
DTA	0.307 * (1.75)	−0.110 ** (−2.25)	0.369 ** (2.04)
NTA	1.100 *** (7.93)	−0.077 ** (−1.99)	1.144 *** (8.06)
asset	0.792 (0.32)	4.667 *** (5.93)	−1.858 (−0.67)
ROA	0.471 (1.38)	0.321 *** (2.96)	0.288 (0.85)
Constant	0.576 (0.01)	−55.670 *** (−3.05)	32.193 (0.50)
Time fixed effect	Controlled	Controlled	Controlled
Province fixed effects	Controlled	Controlled	Controlled
Industry fixed effects	Controlled	Controlled	Controlled
Obs.	765	765	765
$R^{2}$	0.1886	0.1578	0.2054

Note:“ robust T-statistic” is enclosed in parentheses; *, **, and *** denote the levels of significance by 10%, 5%, and 1%, respectively.

## Data Availability

Data was obtained from Trucost, Wind, Eastern Wealth Choice, China Stock Market & Accounting Research Database (CSMAR) and Wharton Research Data Services (WRDS).
